# The Introduction of a Full Medication Review Process in a Local Hospital: Successes and Barriers of a Pilot Project in the Geriatric Ward

**DOI:** 10.3390/pharmacy6010021

**Published:** 2018-02-28

**Authors:** Lies De Bock, Eline Tommelein, Hans Baekelandt, Wim Maes, Koen Boussery, Annemie Somers

**Affiliations:** 1 AZ Oudenaarde, Pharmacy, Minderbroedersstraat 3, 9700 Oudenaarde, Belgium; lies.debock@azglorieux.be (L.D.B.); hans.baekelandt@azoudenaarde.be (H.B.); 2 Pharmaceutical Care Unit, Faculty of Pharmaceutical Sciences, Ghent University, Ottergemsesteenweg 460, 9000 Gent, Belgium; koen.boussery@UGent.be (K.B.); annemie.somer@uzgent.be (A.S.); 3 AZ Oudenaarde, Geriatrics Department, Minderbroedersstraat 3, 9700 Oudenaarde, Belgium; wim.maes@azoudenaarde.be; 4 Department of Clinical Pharmacy, Ghent University Hospital, Corneel Heymanslaan 10, 9000 Gent, Belgium

**Keywords:** medication review, medication reconciliation, inter-professional communication, clinical pharmacy, elderly

## Abstract

For the majority of Belgian hospitals, a pharmacist-led full medication review process is not standard care and, therefore, challenging to introduce. With this study, we aimed to evaluate the successes and barriers of the implementation of a pharmacist-led full medication review process in the geriatric ward at a local Belgian hospital. To this end, we carried out an interventional study, performing a full medication review on older patients (≥70 years) with polypharmacy (≥5 drugs) who had an unplanned admission to the geriatric ward. The process consisted of 3 steps: (1) medication reconciliation upon admission; (2) medication review using an explicit reviewing tool (STOPP/START criteria or GheOP^3^S tool), followed by a discussion between the pharmacist and the geriatrician; and (3) medication reconciliation upon discharge. Ethical approval was obtained from the Ethical Commission of the Ghent University Hospital. Outcomes included objective data on the interventions (e.g., number of drug discrepancies; number of potentially inappropriate prescriptions (PIP)); as well as subjective experiences (e.g., satisfaction with service; opinion on inter-professional communication). There was a special focus on communication aspects within the introduction of this process. In total, 52 patients were included in the study, taking a median of 10 drugs (IQR 8–12). Upon admission, 122 drug discrepancies were detected. During medication review, 254 PIPs were detected and discussed, leading to an improvement in the appropriateness of medication use. The satisfaction of community pharmacists concerning additional communication and the satisfaction of the patients after counselling at discharge were positive. However, several barriers were encountered, such as the time-consuming process to gather necessary information from different sources, the non-continuity of the service due to the lack of trained personnel or the lack of safe, electronic platforms to share information. The communicative and non-communicative successes and hurdles encountered during this project need to be addressed in order to improve the full medication review process and to strengthen the role of the clinical pharmacist.

## 1. Introduction

Transitions of care are defined as the movement of a patient from one healthcare provider or setting to another [1]. Subsequent coordination and continuity of care require timely and accurate communication between the different care providers [1,2]. There are many barriers to effective communication, such as the lack of experience, the complexity of healthcare, the distracting nature of healthcare settings, and the lack of standardization [3].

A lack of communication between caregivers during these transfers of care has been shown to be linked to poor patient outcome [3] as well as to medication errors [4]. The process of medication reconciliation guarantees that the medicines the patient should be prescribed match those that are prescribed. By performing reconciliation at each transfer of care, a comprehensive medication list is continuously available and adapted to the current clinical situation [4,5,6,7,8,9]. The comprehensive medication list is not only important to reduce the risk of medication errors but also serves as the starting point for medication review [5].

By performing a medication review, potentially inappropriate medication (PIM) use can be detected. PIMs consist of over-, under-, and misprescribing of drugs [10,11,12]. Inappropriateness can occur on several levels: wrong dose, frequency, modality of administration or duration of therapy, potential drug–drug or drug–disease interaction, no clear evidence-based clinical indication, or the omission of a clinically indicated treatment or prevention [13]. A screening of the medication by the clinical pharmacist using a screening tool can be a valuable aid to routine pharmacotherapy and pharmaceutical care and can be part of a shared decision-making process between physicians and pharmacists [14]. Several explicit (criteria-based) and implicit (judgement-based) methods to screen for inappropriate prescribing in older patients have been published including the Medication Appropriateness Index (MAI) [15,16], the Screening Tool for Older Persons potentially inappropriate Prescriptions/Screening Tool to Alert for Right Treatment (STOPP/START) [14,17], and the Ghent Older People’s Prescriptions community Pharmacy Screening (GheOP^3^S) tool [18]. 

The complete process of medication reconciliation and review is a good example of a clinical pharmacy service, as it can be performed at an unplanned hospital admission. Clinical pharmacy services, in general, aim to provide patient care that optimizes medication therapy and promotes health, well-being, and disease prevention [19]. However, the introduction of clinical pharmacy services both in primary, secondary, or tertiary care has known some difficulties [20]. 

To support all Belgian hospitals to introduce or expand clinical pharmacy services, the government implemented supplementary financing (0.25 full-time equivalents (FTE) per 200 beds with a maximum of 2 FTE) [21]. Moreover, the government wrote an implementation plan focusing on four domains with specific subjects or goals for each year (see Supplementary Table S1) [22]. Besides this (financial) encouragement, the implementation of clinical pharmacy is necessary for the hospital to obtain a quality accreditation label. This accreditation is not mandatory but it is an unbiased proof of the process quality in the hospital [23]. Several standards are relevant for clinical pharmacy services in order to improve patient and medication safety. 

The new resources provided by the government has enabled the hospital described in this study to supplement the team with a clinical pharmacist (0.5 FTE). This study was conducted to explore the possibilities, the difficulties, and the stakeholders’ views on implementing a full medication review process in the hospital setting. We also aim to provide an overview of the communicative contacts during the process. 

## 2. Methods 

### 2.1. Study Design and Participants

This manuscript reports on the (communicative) successes and barriers that were perceived during a prospective interventional study that was carried out from March to September 2016. Patients were eligible if they (1) had an unplanned admission to the geriatric ward; (2) were at least 70 years old; (3) took at least five drugs chronically at the time of admission; (4) were not hospitalized in the preceding 3 months; and (5) did not have any form of cognitive impairment (mini-mental state examination (MMSE) <21/30 or documented confusion as determined by the clinical judgement of the physician or nurse). The study received ethical approval from the Ethical Commission of Ghent University Hospital. All patients provided an informed consent. 

### 2.2. Study Setting

The study was carried out in the General Hospital in Oudenaarde (Belgium), a local hospital with 235 beds and 3 hospital pharmacists. Prior to the study, a number of changes were introduced to enable the minimal conditions to perform clinical pharmacy. A vision on clinical pharmacy was outlined in cooperation with the Pharmacy and Therapeutics Committee. This included access to the full electronic patient file, granted to the pharmacists after approval by the medical board. In 2016, the main focus was shifted to medication reconciliation at the time of admission and discharge. Before the intervention described below, a hospital-wide basic protocol to improve medication reconciliation was developed and implemented in cooperation with the hospital pharmacy.

### 2.3. Intervention

The intervention consisted of an extended medication reconciliation at the time of admission and a medication review by the clinical pharmacist. Both were completed within 72 h of admission. Additionally, a medication reconciliation was performed at the time of discharge. A summary of the methods of communication is shown in Table 1. 

#### 2.3.1. Medication Reconciliation upon Admission

**Figure 1:**
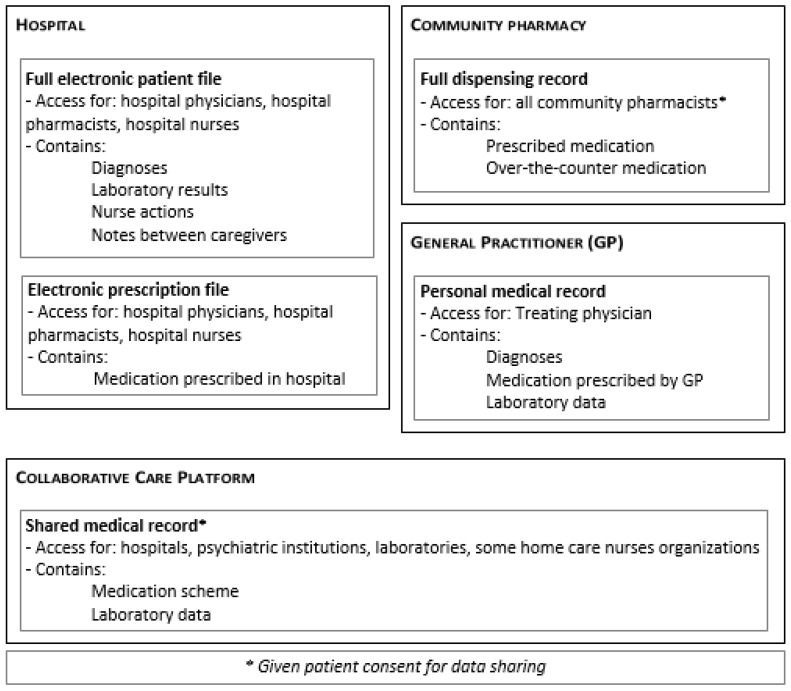
The different patient records consulted during the reconciliation process.

For patients included in the study, the clinical pharmacist obtained the best possible medication history, starting with the initial medication list that was used by the ward upon admission. This initial list was composed by the treating physician with the support of a nurse, following the standard hospital procedure, making use of one or more extra sources including (1) the electronic prescription file if the patient was previously admitted; (2) the shared medical record from the collaborative care platform; (3) the referral letter of the GP, if applicable, and (4) the patient interview at admission. Subsequently, the full electronic patient file was consulted by the clinical pharmacist and discrepancies were noted. A structured interview with the patient or caregiver was performed in order to clarify the current drug use. For each drug included in the medication list, the current use, dosage, and time of administration were checked. Additionally, the limited questions list—which was proven they could reveal omissions [24]—and questions on previous adverse drug events or allergies were discussed. Subsequently, a telephone interview was performed with the community pharmacist in order to obtain the full dispensing record for the preceding 6 months. In Belgium, for chronic patients it is advised that besides all prescribed medications, all dispensed over-the-counter (OTC) medications are registered. As well, dispensing records are shared among all community pharmacies. No OTC medication is dispensed outside of community pharmacies. In some cases, it was necessary to contact the general practitioner (GP) to obtain information from his personal medical record (Figure 1).

The treating physician was informed of every discrepancy between the initial medication list and the best possible medication list. Based on the current status of the patient, the treating physician adjusted the electronic prescription in order to provide optimal therapy. The detected discrepancies were classified as follows: (a) omission on initial list; (b) patient does not take this drug; (c) wrong dose; (d) wrong modality of administration (route/time of administration); (e) wrong formulation; (f) relevant information on duration of therapy; and (g) allergy previously not registered in file. 

Additionally, the contacted community pharmacists were questioned about (1) the presence of a medication list; and (2) their opinion on this inter-professional communication between pharmacists in primary and secondary care.

#### 2.3.2. The Medication Review Process

For the medication review process, patients were allocated to three different groups based on ward and treating physician. The first group consisted of patients treated by a geriatrician on one ward and these patients’ medication lists were screened with the STOPP/START criteria [14,17]. The patients in the second group were treated by the same geriatrician but on the other ward and were screened with the GheOP^3^S tool [18]. Patients who were treated by another internal medicines physician were allocated to the third group, the control group, in which no screening was performed. The medication screening was performed by the clinical pharmacist (LDB) and was based on a pharmaceutical patient file specifically drawn up for the study. This file included (a) the best possible medication history; (b) the current and previous prescriptions in the electronic prescriptions program; (c) relevant adverse drug reactions that occurred in the past; (d) relevant medical history and diagnosis; and (e) information on the current admission, including diagnosis and laboratory results. Although most information was present in the full electronic patient file, it was unstructured. Information could be added to the electronic record, could be in ‘free-text notes’, or in separate pdf documents (i.e., reports from previous admissions or consults within the hospital). We measured the time needed to compose this file. Detected items were first clinically judged by the clinical pharmacist (e.g., check serum potassium when an ACE inhibitor and a potassium-sparing agent were combined), and recommendations for relevant items were sent to the geriatrician by internal e-mail. The geriatrician adjusted prescriptions when needed and an additional meeting between the geriatrician and the pharmacist was set up once a week to discuss recommendations and acceptance. 

In order to estimate the impact of the screening, the adapted MAI (aMAI) [15,16] was calculated by the clinical pharmacist (LDB) and an independent reviewer (ET) in a post-hoc analysis. Medication use at both admission and at discharge were evaluated. A low or high aMAI score suggests appropriate or inappropriate use of the medication, respectively. 

#### 2.3.3. Medication Reconciliation upon Discharge

Upon discharge, the clinical pharmacist reviewed the discharge medication list by comparing it with the medications upon admission, changes during hospital stay, and instructions mentioned in the discharge letter. Unintended discrepancies between the different sources were discussed with the geriatrician and incorrect sources were adjusted in order to avoid confusion in the future. Immediately before discharge, the clinical pharmacists discussed the discharge medication with the patient or caregiver. Changes were emphasized, such as starting, changing, or ceasing medication. The necessary counselling was provided for new drugs, such as administration instructions (with/without meal, time of administration), alarm symptoms of frequent adverse drug reactions, or possible important interactions with food or other drugs. At the end of this counselling, the patients or caregivers were asked if this extensive information session was helpful in understanding the discharge medication list. Moreover, to evaluate the effect on transfer between settings, a pharmaceutical discharge letter was sent to half of the general physicians and community pharmacies of the included patients. This letter contained the discharge medication list, as well as details on the changes compared to the prior medication list. During a follow-up phone call to the receiving general physicians (GP) and community pharmacists, the added value of the pharmaceutical discharge letter was discussed. Three questions were asked: (1) Do you think this letter is useful? (2) What actions do you undertake upon receiving the letter (read, read and file, don’t read)? (3) Do you have suggestions to improve this clinical pharmacy activity? 

### 2.4. Experiences

A special focus was placed on the different needs for communication within every step of the process. Upon initiation of the pilot project, all internal stakeholders (pharmacist, physician, nurses, etc.) were asked to gather their impressions and experiences (both positive and negative) during the process. These experiences were shared during the other contacts with the clinical pharmacist. Moreover, the professionals were asked to provide ideas on how the performed clinical pharmacy activities could be improved. A short report was written by the clinical pharmacist after every contact that contained relevant information on the process and the impressions of the people involved. 

### 2.5. Analysis and Outcomes

This project aimed to identify the successes and barriers encountered during the implementation of a full medication review process as an example of a clinical pharmacy service in a hospital. The primary outcomes were number and type of medication discrepancies at admission and the improvement of prescribing appropriateness. All other outcomes were considered secondary. Both were evaluated and analyzed by the pharmacist in a descriptive way. Descriptive statistics are displayed as counts with percentages, median with interquartile ranges (IQR), and means with standard deviations (SD) as appropriate. Information about experiences is reported qualitatively.

The combination of the results of the interventions and the experience of all participants, both patients and healthcare workers, resulted in a summarizing box for each step of the project, divided into three parts: (1) successes; (2) barriers; and (3) recommendations for the different involved partners. 

## 3. Results 

### 3.1. General

In total, 52 out of 261 (20%) admitted patients were included in this study. The other 80% of patients were excluded based on the first exclusion criteria following this cascade: (1) age < 70 (*n* = 20); (2) taking <5 drugs at home (*n* = 38); (3) admission to a geriatric ward less than 3 months ago (*n* = 40); (4) cognitive impairment (*n* = 94), or (5) absence of the clinical pharmacist or no consent (*n* = 17), and received standard care. 

### 3.2. Medication Reconciliation at Time of Admission

Fifty-two medication reconciliations were performed. The final medication lists totaled 529 drug or food supplements (median of 10 per patient (IQR 8–12)). In total, 122 discrepancies (122/529; 23%) were detected; 58 (11%) were obtained or altered after the structured interview with the patient or caregiver (median of 1 per patient (IQR 0–2)) and 64 (12%) after consultation with the community pharmacist (median of 1 per patient (IQR 0–2)). For 10 patients (19%), no community pharmacy could, however, be consulted (patients living in a nursing home, family member chooses community pharmacist, or patient did not have a regular community pharmacist). For some patients, the general physician (GP) was contacted when relevant information was missing. These communications often required multiple attempts due to inaccessibility of the GP or patient file while the GP was performing home visits. 

The types of discrepancies are depicted in Table 2. Supplementary Table S2 shows the anatomical and therapeutic classes of the drugs that were involved in the 122 items recovered or altered after patient/caregiver or community pharmacist interview, as well as some examples. 

Every contacted community pharmacist (*n* = 20, some patients visited the same community pharmacy) was positively surprised with the request for additional information and considered this contact positive. For only two patients, the community pharmacist said a medication list was available. One pharmacist mentioned that she asks patients with complex schemes whether they desire one and often (9/10) patients say they do not want or need one. 

The process to obtain the best possible medication history was time consuming with patient interviews sometimes taking over 20 min, or due to the need for multiple attempts to contact the pharmacists or GPs.

### 3.3. The Medication Review Process

The creation of a useful and clear pharmaceutical patient file took an average (±SD) of 23.5 (±10.5) min. The subsequent mean (±SD) time needed to perform the medication review using the GheOP^3^S tool and the STOPP/START criteria was 12.0 (±4.3) and 15.2 (±4.9) min, respectively. A total of 254 PIMs were detected, resulting in 195 therapeutic recommendations for the treating geriatrician after clinical judging by the pharmacist (see Supplementary Table S3). Thirteen percent of the recommendations was fully accepted and 6% was partially accepted (e.g., different dose, different alternative drug). Incorrect screening by the pharmacist was observed due to incompleteness of the patient file at the time of consultation by the clinical pharmacist or due to misinterpretation or mistakes by the pharmacist. There was an improvement in the aMAI scores for 75% and 88.2% of the patients after screening with the GheoP³S tool (*n* =20) or STOPP/START criteria (*n* = 17), respectively. In the control group (*n* = 9), this improvement was seen in only 55.6% of patients (Table 3). Due to the small sample size of this study, it was not possible to perform statistical analysis on the results. However, a clear trend towards an improvement of prescribing appropriateness, as expressed by a decrease in aMAI, was observed with both the GheOP³S tool and the STOPP/START criteria.

### 3.4. Medication List at Discharge and Pharmaceutical Discharge Letter

A total of 46 of the included patients were discharged within the study period. Six patients were lost in follow-up due to death during admission or discharge beyond the study period. Forty-one medication lists were evaluated by the pharmacist upon discharge, as 5 patients were discharged during the absence of the participating pharmacist. Overall, there was a good agreement between the medication list and the information in the discharge letter from the physician. The most important detected non-agreements were the omission of warfarin on a scheme and the wrong dose of pantoprazole. Other interventions by the pharmacist were the substitution to the brand or generic drug used at home from a different brand or generic drug during hospitalization or the adjustment of modalities of administration (e.g., time of administration, need to be sober). Every patient with whom the medication list was discussed evaluated this intervention positively.

For 24 patients, a pharmaceutical discharge letter was sent to the general physician and community pharmacist. Seventeen of the 18 pharmacists thought the information in this letter was useful and saved the information in the pharmacy software or patient file. Only 7 of the 16 general physicians were positive. Main reasons to not support this were (a) information is already available in the discharge letter; (b) unnecessary with the anticipation of an upcoming shared electronic patient file; (c) additional administrative burden (another letter to read and file).

## 4. Discussion

We performed a prospective interventional study, implementing a full clinical pharmacy service in a local Belgian hospital. The service consisted of medication reconciliation at the time of admission, a medication review, and a medication reconciliation at the time of discharge. Fifty-two patients were included. A discrepancy was detected for about one in four drugs. Medication review with a validated tool improved medication appropriateness according to the MAI score. A discharge consultation with the clinical pharmacist was positively evaluated by the patients. 

### 4.1. Medication Reconciliation at Time of Admission

The results show a positive influence of a pharmacist-led medication reconciliation upon admission, which is in line with many previous studies [7,8,25]. It also reflects the importance to consult more than one source to obtain information on the patient’s medication history [5]. The number of detected discrepancies in this study through the communication with the community pharmacist is probably an underestimation of the actual possible gain of information, as the community pharmacist could not be contacted for about one-in-five patients. Difficulties to obtain the best possible medication history following this protocol are expected when patients with cognitive impairment are admitted, as they most likely will lack knowledge on their current and previous drug use, as well as on their community pharmacy. 

Many of the detected discrepancies led to changes in the prescriptions during the first days of admission and thus decreased the risk of therapeutic failure or medication errors. Some of the omissions concerning (non-)prescription drugs provided valuable information on additional symptoms experienced by the patient, as these symptoms could be signs for adverse drug reactions (e.g., fall incident with head wound after recent initiation of tramadol/paracetamol; or the need for chronic laxatives or artificial tears due to long term combination of drugs with anticholinergic properties). Additionally, the use of, for example, calcium supplements at home is relevant due to the possible influence on the absorption of several drugs. In contrast, some of the discrepancies were not taken into account by the physician in his evaluation of the prescriptions, such as the omission of multivitamins or homeopathic preparations. 

As communication between the pharmacist and geriatrician was performed by telephone call or by e-mail, the only trace was the adjustments by the geriatrician in the electronic prescriptions. In order to improve traceability of the activities and interventions, as well as to provide all known information to every healthcare provider of the patient, future projects would benefit from registration in the electronic patient file. Ideally, this should be done in a section specifically designated for pharmacotherapeutic information.

The request for information on the medication dispensing history was positively experienced by the community pharmacists. Community pharmacists have a strong therapeutic relationship with patients and have access to the full dispensing records. Community pharmacists should use this important qualification in, for example, providing medication lists to patients. They should also inform patients on the helpfulness of an updated medication list in (often unexpected) transitions of care. This key role of the community pharmacist has been recently confirmed by the approval of a multiannual framework in Belgium [26]. Patients with chronic conditions will be able to choose a family pharmacist to help them in the follow-up of their treatment. The family pharmacist can help by providing the patient with an up-to-date medication list and supporting the patient in obtaining maximal therapeutic adherence. Compared to the easy and fast communication with the community pharmacists, contact with the general physicians was more difficult. We often needed multiple attempts; however, the required information was always obtained within a reasonable period.

An important barrier to implement a thorough medication reconciliation as standard care is the time to perform this task. Obtaining the best possible medication history through the above described procedure requires staff who are sufficiently trained to perform this task, possibly a pharmacy technician. Moreover, it is a time-consuming process and staffing is not equal 24/7, making it difficult to provide this service on a regular basis. However, the process of medication reconciliation could be facilitated through a centralized (pharmaceutical) patient file, which creates the possibility to share information between healthcare providers and settings. The Virtual Integrated Drug Information System (VIDIS) platform is being developed by the Belgian government for this purpose but is not yet implemented in every hospital or pharmacy and is, therefore, not yet usable. Until the implementation of this platform, the need for an extensive process of medication reconciliation remains. A summary of the successes, barriers and recommendations concerning medication reconciliation upon admission can be found in Table 4. 

### 4.2. The Medication Review Process

The current software system used in the hospital is not sufficiently integrated to facilitate the creation of a useful pharmaceutical file consisting of crucial patient diagnostic, laboratory, and prescribing (history) information. The need to consult many different sources increases the risk of missing relevant items. Moreover, gathering the information takes a lot of time and decreases the number of patients for whom a medication review can be executed. A clear, uncomplicated patient file is again a must-have in order to perform medication reviews in a timely manner. This integrated patient file should be available for every professional with a therapeutic relationship with the patient. Also, physicians and other healthcare workers should be motivated to keep the file up to date and to make sure that everyone who is involved in the treatment of the patient is sufficiently informed.

The time needed to perform a medication review and to formulate the recommendation was comparable for both tools. This shows that when a clear patient file is present, medication review can be performed within a reasonable amount of time. The adjustment of about 20% of the potential inappropriate prescriptions depicts the potential role a clinical pharmacist can play in the optimization of the medication list.

In most cases, the geriatrician read the internal e-mail containing the recommendations; however, some were missed and recommendations were only evaluated during the weekly meeting. In the case of acceptance of the recommendation, this could lead to a delay in therapeutic change of up to 7 days. To avoid these unnecessary and potentially harmful delays, adjustments in communication strategy are necessary. For example, when recommendations are communicated through e-mail, reading confirmations should be asked for. Moreover, everything should be registered in the patient file, readily available for the physician, and a system should be set up to notify the physician that recommendations are present. A more intense follow-up with a reminder for the physician after a certain amount of time could also be beneficial. 

Another way to improve communication is the presence of the clinical pharmacist during multidisciplinary meetings. As such, medication-related issues could be addressed on a regular basis. The pharmacists can also be present on the ward or participate in ward rounds in order to provide immediate pharmacological support, a strategy that has been proven to be successful [27,28,29]. Table 5 summarizes the successes and barriers encountered during this part of the project, as well as shows the recommendations to the different involved partners. 

### 4.3. Medication List at Discharge and Pharmaceutical Discharge Letter

Discharge is another important transfer of care that requires specific attention. The patient or caregiver should be informed on the correct therapeutic plan to follow after the hospitalization, which starts with a correct medication list. Evaluation of the scheme by the clinical pharmacist showed that the majority of the schemes were concordant with the actual therapeutic plan of the physician, as noted in the patient file and the discharge letter. However, the detection of some errors confirmed the usefulness of a review, as in rare cases important drugs were omitted or the dosage was incorrect. Additionally, the frequent need to substitute some drugs to the brand the patient was using prior to hospitalization is an important intervention as this could lead to confusion or even double intake of the same drug. The preparation of the medication list prior to discharge is a condition to be able to perform this clinical pharmacy activity. During the study, the participating geriatrician always provided the scheme on time. The software supporting this activity was, however, evaluated to not be user friendly, keeping other physicians from preparing a medication list. The participation of only one clinical pharmacist in the project caused the service of the evaluation of the discharge medication list to not be continuous.

Due to the lack of a safe electronic system used by every healthcare provider to share patient information, the pharmaceutical discharge letters were sent by conventional mail. Community pharmacists reacted overall very positively to the pharmaceutical discharge letter. They believed they could provide better pharmaceutical care for their patient as they could inform the patient on the changes. The therapeutic plan was also clearer for the pharmacist him/herself, which made them more confident they were dispensing the correct drugs. General physicians’ opinions on the pharmaceutical discharge letter were divided. The reason to support the use of this letter was the elimination of the need to search the entire discharge letter for scattered information on pharmacotherapeutic information. A summary of the experiences and recommendations on medication reconciliation upon discharge is provided in Table 6.

## 5. Strengths and Limitations

To our knowledge, this is the first study to provide an overview of the communicative contacts during a full medication review process by a clinical pharmacist. The successes and barriers were analyzed from both a quantitative and qualitative perspective, taking into account the opinions of every involved participator in the process. Additionally, the large amount of information and experiences were used to formulate recommendations, which could support the short- or long-term improvement of the implementation or expansion of a full medication review process. 

The recommendations described in the study could apply to different patient populations, as medication reconciliation or medication review are important processes for every patient undergoing a transition of care, taking multiple drugs, or with a complex pathology. Moreover, the results from the study could be beneficial for other types of institutions, such as nursing homes, as they also often struggle with the same barriers. 

Nevertheless, this study has some limitations. The small number of included patients precluded the statistical comparison of the groups, resulting in only descriptive statistics. This project shows there is a need to include patients with cognitive impairment in studies evaluating the effect of medication screening tools, as they represent a major part of geriatric patients requiring hospitalization. Moreover, the positive effect of screening on medication appropriateness, as reflected in improvement of outcome parameters such as MAI score, cannot be extrapolated to a positive effect on other outcomes, such as drug reactions, drug-related hospital admissions, healthcare use, patient reported outcomes, and mortality [30,31]. These parameters are affected by many interventions and not only by the medication review by the pharmacist. There is, however, not yet a consensus on how to evaluate the effect of a medication review as the outcome reporting from published trials is heterogeneous [30]. Therefore, future trials evaluating the effects of pharmacist-led medication reviews should use a core outcome set (i.e., an agreed standardized collection of outcome variables that should be measured and reported in all trials for a specific clinical area [30]). 

Another limitation was the participation of only one clinical pharmacist and one geriatrician, compromising the continuity of this clinical pharmaceutical service. For the pharmacists, the problem is dual: (1) a shortage of staff to provide continuous service; and (2) the lack of training of the hospital pharmacists to perform this task. Shortage of staff is, however, being partially addressed by the Belgian government through structural financing. Nevertheless, the level of knowledge of this subject in Belgian hospital pharmacists should improve in the upcoming years as the training of hospital pharmacists has been prolonged (from 1 to 3 years), with additional focus on clinical pharmacy (theoretical course + training program), and hospital pharmacists could further specialize in clinical pharmacy by following additional courses [23,32]. Informing physicians (in training) on the positive influence of a clinical pharmaceutical intervention could increase their enthusiasm to collaborate.

The experiences of the involved healthcare workers were collected by the same clinical pharmacist who was involved in the execution of the clinical pharmacy service, in reports of the often verbal inter-professional contacts. As this could possibly introduce bias in the obtained results, future studies could benefit from written questions or independent interviewers. Additionally, a formal analysis with focus groups is needed; however, this was beyond the scope of this implementation study.

## 6. Future Perspectives

Clinical pharmacy services can only be effective if the treating physician uses the results to optimize patient treatment. Therefore, the results from the pilot project will be reported to the medical staff of the hospital in order to create awareness on the potential role of clinical pharmacy for their patients and to stimulate their enthusiasm. After performing and evaluating this pilot project, we are ready to introduce front-office clinical pharmacy at our local hospital. Every project should be preceded by adequate training of the involved pharmacists and other healthcare workers, as well as by the definition of a clear communication plan. 

The differences in expectations on inter-professional communication between pharmacists and GPs have been recently described for the German situation by Weissenborn et al. [33]. A similar study, performed on a local or national level in Belgium, could be helpful in the development of means of communication, including standardized recommendations, suitable for every professional.

As demonstrated in the analysis of our pilot project, further extension of clinical pharmacy, in general, will need optimization on different levels. Healthcare providers should receive intensive training and should recognize each others’ roles in the optimization of medication and patient safety. Interdisciplinary and seamless healthcare can be facilitated by the creation of structured patient files that are easily and securely accessible by every professional and with the possibility to communicate. This should be developed both locally (within the hospital) and externally through the VIDIS platform currently under development. 

## 7. Conclusions

The introduction of clinical pharmacy should be considered in every hospital as this has a significant impact on patient safety and patient care. The importance of a prominent role of the clinical pharmacist in the optimization of drug use in older people through the implementation of a full medication review process has been shown in this study. However, opposite every success, several barriers were detected or perceived, showing the difficulties in setting up a structure to be able to perform clinical pharmaceutical activities. Clear inter-professional communication has been shown to be essential during every step in order to obtain positive results and to facilitate the process. The identification and analysis of the communicative and non-communicative successes and hurdles encountered during this project need to be addressed in order to improve the full medication review process.

## Figures and Tables

**Table 1:** Summary of the actions and corresponding methods of communication within each step of the intervention.

Source of Information	Action	Means of Communication
Medication reconciliation upon admission		
Patient file	Obtain initial list of medication	Electronic consultation
Patient	Structured interview	Face-to-face
Community pharmacist	Request dispensing history	Phone
GP (if needed)	Request medication history, clarification	Phone
Treating physician	Report and discuss discrepancies	Phone, internal e-mail
Medication review		
Patient file	Obtain data needed for screening	Electronic consultation
Treating physician	Discuss results of the medication review	Internal e-mail within 72 h upon admission and weekly face-to-face discussion
Medication reconciliation upon discharge		
Patient file	Review of the discharge medication list	Electronic consultation
Treating physician	Discuss discrepancies	Face-to-face, phone, internal e-mail
Patient	Discuss discharge medication list	Face-to-face
GP	Provide pharmaceutical discharge letter	Mail
Community pharmacist	Provide pharmaceutical discharge letter	Mail
Follow-up		
GP	Discuss pharmaceutical discharge letter	Phone
Community pharmacist	Discuss pharmaceutical discharge letter	Phone

**Table 2:** Types of detected discrepancies during medication reconciliation upon admission.

Types of Discrepancy	*n* (%)
Total	122
Omission on initial list	83 (70%)
Patient does not take drug	19 (16%)
Wrong dose	14 (12%)
Wrong modality (route/time of administration)	2 (2%)
Allergy previously not registered in file	2 (2%)
Wrong formulation	1 (0.5%)
Information on duration of therapy	1 (0.5%)

**Table 3:** Changes in MAI scores from admission to discharge.

		GheOP^3^S	STOPP/START	Control
Number of patients upon discharge		20	17	9
Patients with improvement in MAI score	*n* (%)	15 (75%)	15 (88.2%)	5 (55.6%)
Patients in whom MAI score stayed equal	*n* (%)	4 (20%)	1 (5.9%)	3 (33.3%)
Patients with deterioration in MAI score	*n* (%)	1 (5%)	1 (5.9%)	1 (11.1%)

GheOP^3^S: Ghent Older People’s Prescriptions community Pharmacy Screening; STOPP/START: Screening Tool for Older Persons potentially inappropriate Prescriptions/Screening Tool to Alert for Right Treatment.

**Table 4:** Box 1: Medication Reconciliation upon Admission.

**Successes** Identification of a high number of discrepancies after the consultation of multiple sources Identification of additional symptoms or adverse reactions through medication use Communication with community pharmacy was appreciated by both parties	**Barriers** Lack of patient knowledge (whether or not due to cognitive impairment) Confusion due to discrepancies between sources No registration of medication reconciliation in patient file, no separate pharmaceutical section Accessibility of general physician Time consuming, 24/7 serviceLack of an integrated (pharmaceutical) centralized patient file
**Recommendations for** Patients: always bring an up-to-date medication list, including non-prescription medication Hospitals: provide a protocolled medication reconciliation procedure, including registration in patient file and train healthcare providers to perform them Community pharmacists: provide a clear medication list for the patient, including OTC drugs; encourage patients to keep medication list up to date Software: develop/provide a section for pharmaceutical interventions in patient file Government: facilitate transfer of information (centralized patient file), fund community and clinical pharmacists to improve and expand seamless communication of drug use	

**Table 5:** Box 2: Medication Review and Therapeutic Recommendations.

**Successes** Full access to patient file Relatively fast screening with the tools Identification of a significant amount of PIMs Improvement in prescribing appropriateness (MAI scores) 20% of therapeutic recommendations were accepted	**Barriers** Scattered information across different programs: risk of incomplete pharmaceutical file + time consuming Continuity of presence of pharmacist(s) and participating physician(s) Incorrect screening by the pharmacist Inefficient communication Agreements prior to interventions on what to recommend (service level agreement)
**Recommendations for:** Patients: empower patients: they should be involved in decisions Hospitals: provide access to complete files for clinical pharmacist; integrate the presence of pharmacists in multidisciplinary meetings/ward rounds (in order to increase insight into patient condition); encourage and support multidisciplinary meetings Government: financing of sharing files and the execution of medication reviews Training: improve training of physicians and pharmacists to perform medication reviews and to improve communication ICT + research: improve electronic patient file management, create automatization of screening, develop clinical decision support systems	

**Table 6:** Box 3: Medication Reconciliation upon Discharge.

**Successes** Informing patients on medication list upon discharge (pharmaceutical care!) Pharmaceutical discharge letter Extra information for community pharmacist	**Barriers** Software not user friendly to prepare scheme Time-consuming process Communication with other healthcare providers: mail versus safe electronic system
**Recommendations for:** Patients: inform them about the possibilities Hospitals: implement structural discharge consultation with clinical pharmacist; increase awareness of physicians for the importance of a medication list Government: support development of safe ways for communication (VIDIS); increase funding for clinical pharmacy activities ICT: facilitate the preparation of the medication list upon discharge	

## Supplementary Materials

The following are available online at http://www.mdpi.com/2226-4787/6/1/21/s1, Table S1: Domains and themes in the action plan of the Belgian Government (2015–2020); Table S2: Anatomical and therapeutic classes of the drugs involved in the discrepancies detected after medication reconciliation; Table S3: Detected PIMs with the GheoP^3^S tool (a) and the STOPP/START tool (b).
