# Supplementary material for: The Introduction of a Full Medication Review Process in a Local Hospital: Successes and Barriers of a Pilot Project in the Geriatric Ward
Source: Pharmacy (Basel). 2018 Feb 28;6(1):21. doi: 10.3390/pharmacy6010021 (PMC5874560; doi:10.3390/pharmacy6010021)
Supplement: Supplementary file 1 [file pharmacy-06-00021-s001.pdf]

A SUPPLEMENTARY TABLES

Table S1: Domains and themes in the action plan of the Belgian Government (2015-2020)

Domains:
1. Optimizing medication use, using front- and back office strategies
2. Continuity of pharmacotherapy in light of transmural care
3. Scientific skills of the hospital pharmacist
4. Transfer of information to and communication with the patient

Themes:
2015: anchoring the minimal conditions for the application of clinical pharmacy
2016: development of a structured method for the anamnesis, registration and communication of the medication on admission and discharge
2017: application of clinical pharmacy for specific therapies
2018: application of risk screening for patient groups
2019: application of risk screening for medication groups or pathologies
2020: evaluation of the development of clinical pharmacy in the Belgian hospitals and evaluation of the action plan 2015-2020

Table S2: Anatomical and therapeutic classes of the drugs involved in the discrepancies detected after medication reconciliation

	n (%)	Example(s) of discrepancy
Gastro-intestinal system	***43 (35.2%)***	
antacids	6	O: antacid PRN
antihistaminics	1	O: ranitidine 150 mg 1 pd
proton pump inhibitors	5	O: pantoprazole 20 mg 1 pd
propulsives	3	O: domperidone PRN; N: alizapride
laxatives	9	O: macrogol PRN or 1 pd, bisacodyl PRN or 1 pd
antipropulsives	2	O: loperamide 1 pd
probiotics	1	O: frequent need for probiotics
antidiabetics	1	D: dose repaglinide unknown
multivitamins	5	O: multivitamins 1 pd
vitamin D	5	O: vitamin D 1 per week
vitamin B	2	N: vitamin B complex
Calcium/Vitamin D	3	O: Calcium/vitamin D, F: chew tablet (not effervescent tablet)
Nervous system	***26 (21.3%)***	
analgesics	18	O: paracetamol 1 g PRN or 3 pd
benzodiazepines	2	N: lormetazepam 1 mg 1 pd
antidepressants	6	O: duloxetine 60 mg 1 pd, M: mirtazapine 15 mg at 20:00 (not 08:00)
Blood and cardiovascular system	***17 (13.9%)***	

antithrombotics	3	O: fenprocoumon; D: rivaroxaban 15 mg (not 20 mg)
folic acid	1	D: folic acid 1 mg (not 4 mg)
vasodilators	1	O: molsidomine 16 mg 1 pd
diuretics	5	O: indapamide 2,5 mg 1 pd; N: bumetanide 1 mg 1 pd
beta blockers	2	O: bisoprolol 5 mg 1 pd; D: bisoprolol 5 mg (not 10 mg)
calcium channel blockers	1	O: amlodipine 5 mg 1 pd
combinations	1	D: irbesartan/hydrochlorthiazide 300/50 mg (not 300/12,5 mg)
statins	3	O: simvastatin 20 mg 1 pd
Sensory organs	***9 (7.4%)***	
artificial tears	6	O: daily drops dry eyes
antiglaucoma preparations	2	O: latanoprost eyes drops
nasal decongestants	1	O: nasal spray (framycetine, prednisolone, nafazoline)
Dermatologicals	***8 (6.6%)***	
antifungals	3	O: miconazol 2% spray PRN
antibiotics	1	N: fusidic acid 20 mg/g cream
corticosteroids	1	O: bethamethason valerate 0,1% cream very frequently
anti-inflammatory	3	O: diclofenac 2% cream PRN
Musculo-skeletal system	***4 (3.3%)***	
anti-gout preparations	2	D: allopurinol 300 mg 1 pd (not 100 mg)
bone structure and mineralization	2	O: denosumab 60 mg 1 per 6 months
Respiratory system	***4 (3.3%)***	
corticosteroids	1	O: budesonide 200 µg inhalation 2 pd
mucolytics	1	D: acetylcysteïne 600 mg 1 pd (not 200 mg)
antihistaminics	2	D: cetirizine 10 mf 1 pd (not PRN)
Hormonal system	***2 (1.6%)***	
corticosteroids	1	O: prednisone
antithyroids	1	N: thiamazole
Genito-urinary system and sex hormones	***2 (1.6%)***	
alpha antagonists	1	N: tamsulosine 0,4 mg
other	1	O: fenazopyridine 100 mg 1 pd
Anti-infectives for systemic use	***2 (1.6%)***	
antifungals	1	DU: fluconazole 200 mg 1 per week already for 3 months
other	1	N: nifurtoïnol
Allergy previously not in file	***2 (1.6%)***	phenobarbital allergy (documented by general physician)
Homeopathic preparations	***2 (1.6%)***	
Antineoplastic and immunomodulating agents	***1 (0.8%)***	
antimetabolites	1	M: methotrexate 5 mg subcutaneous weekly (not oral)

D: wrong dose; DU: duration of therapy; F: formulation; M: modality of administration (route, time); N: not on current medication list; O: omission; pd: per day; PRN: pro re nata (if needed)

Table S3: detected PIMs with the GheoP³S tool (a) and the STOPP/START tool

S3(a): detected PIMs with the GheoP³S tool			
**Positive items**		**prevalence n = 87**	**number of patients n (%), n=22**
Part 1: potentially inappropriate drugs, independent of diagnosis			
Item number			
1	Any antidepressant ≥ 1 year	8	7 (32%)
26	Rivaroxaban	5	5 (23%)
5	Any short- or long-acting benzodiazepine	3	3 (14%)
9	Any PPI at full dose ≥ 8 weeks	3	3 (14%)
8	Any oral NSAID	2	2 (9%)
16	Dabigatran	2	2 (9%)
4	Any intermediate acting benzodiazepine or Z-product at full dose or any dose ≥30 subsequent days OR	1	1 (5%)
Add	Sotalol for rate control	1	1 (5%)
Part 2: potentially inappropriate drugs, dependent of diagnosis			
Add	Drugs in renal impairment or congestive heart failure [a]	13	6 (27%)
40	Thiazide and loop diuretics with gout	4	4 (18%)
33	Anticholinergics with known dementia or cognitive impairment	2	2 (9%)
36	Calcium channel blockers with constipation	1	1 (5%)
Part 3: PPOs			
45	The patient has an elevated risk for osteoporosis (determined via FRAX tool) and is not prescribed calcium/vitamin D supplementation	14	14 (64%)
47	The patient did not receive yearly influenza vaccination	8	8 (36%)
Add	Start statin in secondary prevention when a patient has elevated total cholesterol and a good life expectancy	1	1 (5%)
Part 4: DDIs of specific relevance			
65	Oral diabetic/insulin + cardio selective beta blocker	7	5 (23%)
77	Any combination of anticholinergic drugs	4	4 (18%)
50	RAAS inhibitor + potassium sparing diuretic/potassium containing drugs	2	2 (9%)
49	VKA + oral NSAID	1	1 (5%)
54	Oral NSAID + diuretic	1	1 (5%)
61	RAAS inhibitor + oral NSAID	1	1 (5%)
64	Oral diabetic/insulin + non-selective beta blocker	1	1 (5%)
74	Calcium + levothyroxine	1	1 (5%)
75	Bisphosphonate + Ca^{2+}, Mg^{2+}, Zn^{2+}, Fe^{2+} of Al^{3+}	1	1 (5%)

Add: additional screening items not included in initial GheOP³S tool; DDI: drug-drug interaction; NSAID: nonsteroidal anti-inflammatory drugs; PPI: proton pump inhibitor; PPO: potential prescribing omission; RAAS: renin-angiotensin-aldosterone system; VKA: vitamin K antagonist

[a] Drugs in renal impairment (GFR < 50 ml/min): RAAS inhibitors, any potassium sparing diuretic, chlortalidon and thiazides, allopurinol, amoxicillin with full dose clavulanic acid, ciprofloxacin, dabigatran, metformin, nitrofurantoin, norfloxacin, sotalol; drugs in congestive heart failure: verapamil

**S3 (b): detected PIMs with the STOPP/START tool**		
**Positive items**	**prevalence n**	**number of patients n (%), n = 19**
**STOPP criteria**	**135**	
Indication of medication		
Any drug prescribed without an evidence-based clinical indication	51	18 (95%)
Any duplicate drug-class prescription	9	8 (42%)
Any drug prescribed beyond the recommended duration, where treatment duration is well defined	1	1 (5%)
Cardiovascular system		
Beta blocker in combination with verapamil or diltiazem	2	2 (11%)
Loop diuretic as first-line treatment for hypertension	2	2 (11%)
Thiazide diuretic with history of gout or hypo-K, hypo-Na or hyper-Ca	2	2 (11%)
Loop diuretic for the treatment of hypertension with concurrent urinary incontinence	2	2 (11%)
Verapamil or diltiazem with NYHA class III or IV heart failure	1	1 (5%)
Antiplatelet/Anticoagulant drugs		
VKA, direct thrombin inhibitor or factor Xa inhibitor for first pulmonary embolus without continuing provoking risk factors for > 12 months	2	2 (11%)
VKA, direct thrombin inhibitor or factor Xa inhibitor for first deep venous thrombosis without continuing provoking risk factors for > 6 months	1	1 (5%)
Central Nervous system and psychotropic drugs		
Benzodiazepines for ≥ 4 weeks	19	13 (68%)
TCA with dementia, narrow angle glaucoma, cardiac conduction abnormalities, prostatism, or prior history of urinary retention	1	1 (5%)
Acetylcholinesterase inhibitors with known history of persistent bradycardia, heart block or recurrent unexplained syncope or concurrent treatment with drugs that reduce heart rate	1	1 (5%)
First-generation antihistamines	1	1 (5%)
TCA as first line antidepressant treatment	1	1 (5%)
Renal system		
NSAID with eGFR < 50 ml/min	1	1 (5%)
Gastrointestinal system		
PPI for uncomplicated peptic ulcer disease or erosive peptic oesophagitis at full therapeutic dosage for > 8 weeks	2	2 (11%)

Drugs likely to cause constipation where non-constipating alternatives are available	1	1 (5%)
Musculoskeletal System		
Oral bisphosphonate with current or recent history of upper gastrointestinal disease, peptic ulcer disease or upper gastrointestinal bleeding	1	1 (5%)
Urogenital system		
Anticholinergic drugs with dementia, chronic cognitive impairment, narrow-angle glaucoma or chronic prostatism	1	1 (5%)
Drugs that adversely affect those prone to falls		
Benzodiazepines	19	13 (68%)
Neuroleptic drugs	2	2 (11%)
Vasodilator drugs known to cause hypotension in those with persistent postural hypotension	1	1 (5%)
hypnotic Z-drugs	1	1 (5%)
Analgesic drugs		
Oral or transdermal strong opioids as first line therapy for mild pain	2	2 (11%)
Long-acting opioids without short-acting opioids for break-through pain	1	1 (5%)
Anticholinergic drugs		
Concomitant use of 2 or more drugs with anticholinergic properties	7	7 (37%)
**START criteria**	**19**	
Cardiovascular system		
Statin therapy with history of coronary, cerebral, or peripheral vascular disease without contraindication	2	2 (11%)
ACE inhibitor with systolic heart failure and/or documented coronary artery disease	1	1 (5%)
Respiratory system		
Regular inhaled -2 agonist or anticholinergic agent for mild to moderate asthma or COPD	1	1 (5%)
Central Nervous system and Eyes		
SSRI (or SNRI or pregabalin if SSRI contraindicated)in persistent severe anxiety that interferes with independent functioning	1	1 (5%)
Musculoskeletal System		
Calcium/vitamin D supplement in patients with known osteoporosis and/or previous fragility fracture(s)	4	4 (21%)
Vitamin D supplement in housebound patients, patients experiencing falls or patients with osteopenia	6	6 (32%)
Vaccines		
Seasonal trivalent influenza vaccine	4	4 (21%)

ACE: angiotensin converting enzyme; PPI: proton-pump inhibitor; SNRI: serotonin and noradrenalin reuptake inhibitor; SSRI: selective serotonin reuptake inhibitor; TCA: tricyclic antidepressant; VKA: vitamin K antagonist
